# TNF-α Decreases VEGF Secretion in Highly Polarized RPE Cells but Increases It in Non-Polarized RPE Cells Related to Crosstalk between JNK and NF-κB Pathways

**DOI:** 10.1371/journal.pone.0069994

**Published:** 2013-07-29

**Authors:** Hiroto Terasaki, Satoru Kase, Makoto Shirasawa, Hiroki Otsuka, Toshio Hisatomi, Shozo Sonoda, Susumu Ishida, Tatsuro Ishibashi, Taiji Sakamoto

**Affiliations:** 1 Department of Ophthalmology, Kagoshima University Graduate School of Medical and Dental Sciences, Kagoshima, Japan; 2 Department of Ophthalmology, Hokkaido University, Sapporo, Japan; 3 Department of Ophthalmology, Kyushu University, Fukuoka, Japan; Chang Gung University, Taiwan

## Abstract

Asymmetrical secretion of vascular endothelial growth factor (VEGF) by retinal pigment epithelial (RPE) cells *in situ* is critical for maintaining the homeostasis of the retina and choroid. VEGF is also involved in the development and progression of age-related macular degeneration (AMD). We studied the effect of tumor necrosis factor-α (TNF-α) on the secretion of VEGF in polarized and non-polarized RPE cells (P-RPE cells and N-RPE cells, respectively) in culture and *in situ* in rats. A subretinal injection of TNF-α caused a decrease in VEGF expression and choroidal atrophy. Porcine RPE cells were seeded on Transwell™ filters, and their maturation and polarization were confirmed by the asymmetrical VEGF secretion and trans electrical resistance. Exposure to TNF-α decreased the VEGF secretion in P-RPE cells but increased it in N-RPE cells in culture. TNF-α inactivated JNK in P-RPE cells but activated it in N-RPE cells, and TNF-α activated NF-κB in P-RPE cells but not in N-RPE cells. Inhibition of NF-κB activated JNK in both types of RPE cells indicating crosstalk between JNK and NF-κB. TNF-α induced the inhibitory effects of NF-κB on JNK in P-RPE cells because NF-κB is continuously inactivated. In N-RPE cells, however, it was not evident because NF-κB was already activated. The basic activation pattern of JNK and NF-κB and their crosstalk led to opposing responses of RPE cells to TNF-α. These results suggest that VEGF secretion under inflammatory conditions depends on cellular polarization, and the TNF-α-induced VEGF down-regulation may result in choroidal atrophy in polarized physiological RPE cells. TNF-α-induced VEGF up-regulation may cause neovascularization by non-polarized or non-physiological RPE cells.

## Introduction

Retinal pigment epithelial (RPE) cells *in situ* play important roles in maintaining the homeostasis of the retina and choroid [1]–[5]. The RPE cells are the major source of vascular endothelial growth factor (VEGF) in the posterior pole of the eye, and they secrete VEGF predominantly on their basal side, i.e., asymmetrical secretion [6]–[8]. The presence of VEGF is important because it is a neuroprotective factor as well as a potent angiogenic factor [9]–[11]. The asymmetrical secretion of VEGF is an important property of healthy RPE cells and is critical for the survival and maintenance of the retina and choroid.

Age-related macular degeneration (AMD) is a leading cause of blindness in older individuals in developed countries [12]. There are two types of AMD, the wet type and the dry type of AMD, and RPE cells are extensively involved in the pathology of both types of AMD. Immunohistochemical analyses have shown that many RPE cells are present in the choroidal neovascular (CNV) membranes that express VEGF [13]–[15]. Earlier *in vitro* studies showed that RPE cells increase their synthesis of VEGF when stimulated by inflammatory cytokines [16], [17]. Thus, they are considered to be accelerators of CNVs in eyes with exudative AMD. However, if inflammation always accelerated angiogenesis, then it would be difficult to fully explain the absence of CNV in the dry type AMD because it is also associated with inflammation [18], [19]. Above all, the regulation of VEGF secretion is complex, and the actual mechanisms controlling the expression of VEGF in RPE cells are not well known [16], [17], [20],[21].

The expression of VEGF by RPE cells has been studied in RPE cell cultures, and the results have contributed to our understanding of how RPE cells are involved in the pathophysiology of retinochoroidal diseases. However, it is difficult to interpret these *in vitro* data because RPE cells are very plastic, and their properties, e.g., polarization and differentiation, change easily depending on the culture conditions [22], [23]. Thus, the results obtained from studies of cultured RPE cells that are not polarized might not necessarily represent the results obtained from RPE cells *in situ*.

A new cell culture method was recently developed in our laboratory that produced RPE cells with properties of RPE cells *in situ*, e.g., asymmetrical secretion of VEGF [24], [25]. These RPE cells should then be more suitable for studying the roles and actions of VEGF in retinochoroidal disease processes.

The purpose of this study was to determine the effects of inflammation on the secretion of VEGF by highly polarized and non-polarized RPE cells in culture. To simulate inflammatory conditions, we exposed RPE cells to tumor necrosis factor alpha (TNF-α), because it is a major pleotropic inflammatory cytokine and is found in high concentrations in eyes with AMD. In addition, macrophages, a potential source of TNF-α, are abundant in the subretinal space of AMD eyes [15], [26], [27]. We shall show that exposure of RPE cells to TNF-α down-regulates the VEGF expression in polarized RPE cells but up-regulates it in non-polarized RPE cells. These findings suggest that there may be different forms of the same retinal disease such as the wet and dry AMD.

## Materials and Methods

### Ethics Statement

All animals were treated in accordance with the Association for Research in Vision and Ophthalmology Statement for the Use of Animals in Ophthalmic and Vision Research. The protocol was approved by the Committee on the Ethics of Animal Experiments of the Kagoshima University (Permit Number: MD08089).

### Retinal Pigment Epithelial (RPE) Cell Cultures

Eyes of 5- to 6-month-old swines were purchased from a slaughterhouse (Kagoshima Shokuniku Center, Kagoshima, Japan) and used for the experiments with permission from the slaughterhouse and Kagoshima University. The RPE cells were isolated from the eyes as described in detail [24], [25]. The cells were cultured in alpha modified Eagle’s medium with 2 mM L-glutamine, 100 U/mL penicillin, 100 µg/mL streptomycin (Sigma-Aldrich, St Louis, MO) and 10% fetal bovine serum (FBS; Omega, Tarzana, CA) at 37°C under 5% CO_2_. The cells were grown in this medium containing FBS for one week.

To prepare polarized RPE cells, approximately 1.0×10^5^ RPE cells/cm^2^ were seeded onto fibronectin-coated Transwell filters (12 mm internal diameter; 0.4 µm pore size; Transwell; Corning Costar, Corning, NY), and cultured in RPE maintenance medium containing 10% FBS for 2 days and in 2% FBS thereafter for more than 2 weeks. To prepare non-polarized RPE cells, 1.0×10^5^ cells/cm^2^ RPE cells were seeded onto 12-well plates (Falcon, Becton Dickinson & Co., Lincoln Park, NJ) and cultured with 10% FBS. Polarized and non-polarized cells were used after they attained more than 90% confluence in all experiments.

### Measurement of Transepithelial Resistance (TER)

The TER of the RPE cells on the Transwells filters was measured with the Epithelial Voltohmmeter 2 (EVOM2; World Precision Instrument, Sarasota, FL) as described in detail [24], [25]. The final resistance per unit area (Ω·cm^2^) was obtained by multiplying the TER by the effective growth area.

### Cell Exposures

The RPE cell culture medium was switched to FBS-free medium overnight to minimize the effect of serum. To study the role of specific intracellular signaling pathways, different inhibitors of the pathways were added to the cells 60 minutes before the addition of 10 ng/ml TNF-α (Pepro Tech. Inc., Rocky Hill, NJ). In the experiments with inhibitors, control cells were treated with serum-free medium containing 0.1% dimethyl sulfoxide (DMSO). The inhibitors and their final concentrations used were 20 µM of SB 203580, a p38 MAPK inhibitor, 20 µM of SP 600125, an inhibitor of JNK1,-2, and -3 (Wako Pure Chemical, Osaka, Japan), 10 µM of CAPE, an inhibitor of NF-κB (Calbiochem, San Diego, CA), 10 µM of PD98059, an inhibitor of Erk (Calbiochem), and 25 µM of crucumin, an inhibitor of AP-1 (Sigma-Aldrich). These concentrations were obtained from the results of our preliminary experiments, and from the results of earlier studies [16], [17], [21], [28].

### Enzyme-linked Immunosorbent Assay (ELISA)

The RPE cell culture medium was switched to FBS-free medium overnight to minimize the effect of serum on the treatments. After stimulation with TNF-α for 24 hours, the medium was collected and stored at −80°C until analyses by ELISA. To determine the level of polarized secretion, the concentration of VEGF-A in the upper and lower chambers were measured separately according to the manufacturer’s protocol (Quantikine; R&D Systems, Minneapolis, MN). The amount of VEGF secreted from the polarized RPECs was expressed relative to the total amount of VEGF, viz., the VEGF from the apical side+the VEGF from the basolateral side.

### Semi-quantitative Reverse Transcription–Polymerase Chain Reaction (RT-PCR)

Semi-quantitative RT-PCR was performed as described in detail in an earlier report [29]. Total cellular RNA was isolated from cells using an automated system (QuickGene; Fujifilm, Tokyo, Japan) with reagents contained in a commercial RNA isolation kit (QuickGene RNA Cultured Cell Kit S; Fujifilm). cDNA was synthesized from 1 μg total RNA using random primers purchased from Promega Biotech (Piscataway, NJ), Avian Myeloblastosis Virus RT (AMV RT; Promega Biotech), and Taq PCR core kit (Qiagen, Hilden, Germany). The primers used were:

VEGF forward: GAAGTGGTGAAGTTCATGGA,

VEGF reverse: GCCTTGCAACGCGAGTCTGT);

and

β-Actin forward: CCAGCACCATGAAGATCAAGATC,

β-Actin reverse: ACATCTGCTGCTGGAAGGTGGACA [30].

Semi-quantification of the density of each band was done by Image J software (US National Institutes of Health) with β-Actin as the reference.

### Terminal dUTP Nick-End Labeling (TUNEL) Staining and Cytotoxic Assay

RPE cells were stained by the TUNEL procedure, and a quantification of the number of TUNEL-positive cells was determined by the ApopTag fluorescein direct *in situ* apoptosis detection kit (Chemicon International, Temecula, CA) as described in detail [31]. The number of TUNEL-positive cells in 10 randomly selected microscopic fields (40x) was counted in a masked fashion. The cytotoxicity was also determined with the MTT colorimetric assay kit (Dojin-do, Kumamoto, Japan) according to the manufacturer’s protocol.

### Immunohistochemistry and Transmission Electron Microscopy

The presence of ZO-1, which is a tight junction-associated molecule [24], [25], MCT1, laminin, NF-κB p65, and phospho-c-Jun, which is mediated by JNK, was determined immunohistochemically in polarized and non-polarized RPE cells as described [24], [25]. RPE cells were first permeabolized in phosphate buffered saline (PBS) containing 0.2% Triton X for 30 min followed by fixation in ice cold methanol for 15 min at 4°C. The specimens were blocked in 5% BSA before incubating with each of the primary antibodies: 1∶100 ZO-1 (Invitrogen, Carlsbad, CA); 1∶200 MCT1 and laminin (Abcam Japan, Tokyo, Japan); 1∶50 NF-κB, and 1∶800 phospho-c-Jun (Cell Signaling, Beverly, MA). An anti-rabbit secondary antibody (Alexa Fluor 488 or 594; Molecular Probes, Eugene, OR) was used for 30 minutes in the dark at room temperature. After the immunostaining, the Transwell membranes were dissected with the cells from the inserts with a sharp razor blade and placed on a glass slide. After nuclear staining with DAPI (Vector Labs, Burlingame, CA), the membrane with cells on the slides were observed with an Olympus fluorescence microscope (Olympus, Tokyo, Japan).

For transmission electron microscopy (TEM), the RPE cells were fixed in half-strength Karnovsky’s fixative for 24 hours at 4°C. The fixed cells were processed as described in detail [24], [25]. Sections were examined with a JEOL JEM2100 (Peabody, MA) electron microscope and photographed with an Orius SC1000B Gatan (Pleasanton, CA) digital camera.

### Western Blot Analyses

Western blotting was performed as we described in detail [31], [32]. Briefly, cell lysates were placed under reducing conditions on a 10% Tris-HCl gel (Mini-Protein Tris-Glycine extended precast gels; Bio-Rad, Hercules, CA) and then transferred to PVDF membranes (Thermo Scientific, Rockford, IL). The primary antibody was then applied. After incubation with horseradish peroxidase-conjugated anti-rabbit/mouse secondary antibody, the protein bands were made visible by ECL plus chemifluorescent reagent (GE Healthcare, Piscataway, NJ). Semi-quantification of the density of each band was done by Image J software with Tubulin (polyclonal, Cell Signaling, Danvers, MA) as the reference protein.

### Animal Experiments

Eight-week-old Brown Norway rats (Kyudo, Fukuoka, Japan) were anesthetized by intramuscular injections of ketamine and xylazine. They were injected with 5 ng of TNF-α in 50 μl of 1% sodium hyaluronate (Opegan; Santen, Osaka, Japan) or with 1% sodium hyaluronate (control) into the subretinal region of one eye. Prior to the injection, the anterior chamber was punctured to reduce intraocular pressure as we have described [31].

To confirm the biological effect of TNF-α, a mixture of TNF-α and 10 μg/ml of an anti-TNF-α antibody (R&D Systems) in sodium hyalonate was injected into the subretinal space. The sclera was penetrated at the nasal equator with a 30-gauge needle. Eyes without an injection served as controls. The rats were sacrificed at 24 hours and at day 7 after the injections, and the enucleated eyes were fixed in 4% paraformaldehyde at 4°C overnight. The anterior segment and the lens were removed, and the remaining eye cup was cryoprotected with 10% to 30% sucrose in PBS. The eye cups were then frozen in optimal cutting temperature compound (Sakura Finetech, Tokyo, Japan). Frozen sections were cut at 8 μm, and the sections were dried, blocked with blocking buffer for 1 hour, and processed for hematoxylin-eosin (HE) staining.

Immunohistochemistry was performed using the ABC method (Vectastain ABC kit, Vector Labs) with VIP peroxidase (Vector Labs) as the chromogen. The antibody used for staining was anti-VEGF antibody (Abcam, Tokyo, Japan). Normal rabbit IgG (R&D Systems) was used instead of primary antibody as a negative control in each case.

To investigate the effect of TNF-α on the RPE-choroid complex, the area of the choroid that was exposed or not exposed to TNF-α was compared using ImageJ software. The area of the indicated choroid (a) was divided by the length of the exposed area (b). To avoid the effect of the cutting angle, the area was further divided by the thickness of the sclera at the center of the exposed area (scleral thickness, ST). The value of choroidal area was expressed by:

Area of choroid per unit = 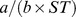.

Three sections which contained the TNF-α or hyaluronan-injected retina were randomly chosen from a single eye. They were evaluated by two masked observers (MS, SS) with no information of the eyes and the average values were used.

### Statistical Analyses

Statistical analyses were performed using SPSS 19.0. All values are expressed as the means ± standard error of the means (SEMs). Differences were considered significant at *P*<0.05. The statistical analyses used were the one-way analysis of variance (ANOVA) followed by post hoc Tukey-Kramer test or Student’s *t* test.

## Results

### Morphological Characteristics of Polarized and Non-Polarized Retinal Pigment Epithelial Cells

**Figure 1:**
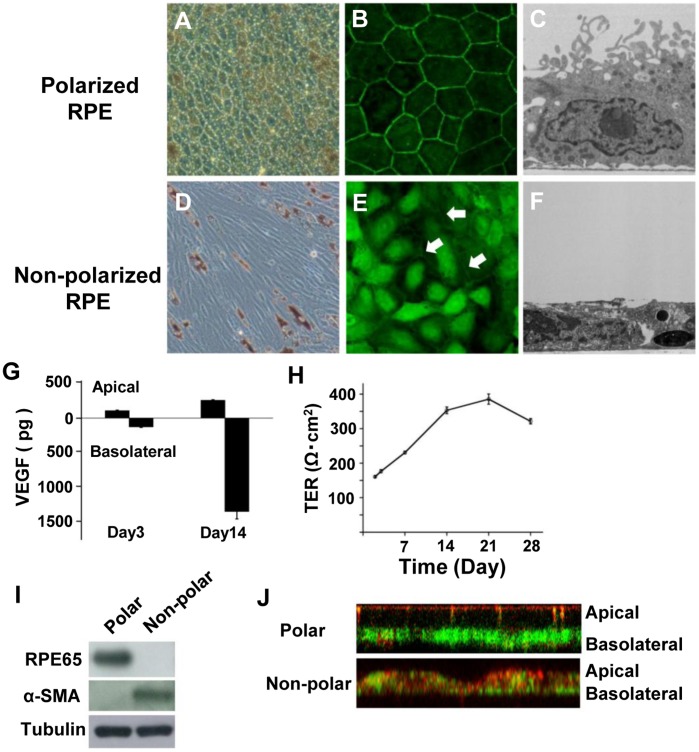
Characteristics of polarized and non-polarized RPE cells. A: B: C: Polarized RPE cells. RPE cells are well pigmented and are arranged in a hexagonal mosaic (A). Intercellular structures outlining each cell are positive to ZO-1 (B). Transmission electron microscopy (TEM) shows that the RPE cells have basally located nuclei and contain pigment granules that congregate close to the apical membrane with well-defined tight junctional complexes. Foot processes on the basal side and numerous microvilli on apical surface can be seen (C). D: E: F: Non-polarized RPE cells. Cells appear spindle- to cobblestone-shaped, and the fine intercellular strands are weakly stained for ZO-1 (E, arrows). TEM shows flat-shaped cells with short microvilli (F). G: Amount of VEGF-A in the incubation media from the upper and lower chambers 3 and 14 days after seeding. On day 3, the amount of VEGF-A secreted from the apical surface was 110.8±8.5 pg/well and the amount was 132.0±6.2 pg/well from the basal surface (*P* = 0.11, paired *t* test). On day 14, the amount of VEGF-A secreted from the apical surfaces was 265.0±7.82 pg/well, and the amount was 1385.9±111.5 pg/well from the basal surface (*P*<0.01, paired *t* test). H: Transepithelial electric resistance (TER) gradually increases over time and reaches a plateau at one month. I: RPE65 is detected by Western blot analysis in polarized RPE cells on day 14 but not in non-polarized cells. α-SMA is detected in non-polarized RPE cells but not in polarized RPE cells. J: MCT1 (red) is located at the apical membrane, and laminin (green) is found on the basolateral side of polarized RPE cells. The staining is more evident in polarized than in non-polarized RPE cells. Original magnifications; A and D: x100, B and E: x200, C and F: x1000.

Fourteen days after seeding the RPE cells on Transwell filters, the cells were well pigmented and were arranged in a hexagonal mosaic (Fig. 1A). At this time, the intercellular structures outlining each cell were positively stained for ZO-1 (Fig. 1B). TEM showed that the RPE cells had basally located nuclei and contained pigment granules that congregated close to the apical membrane as in RPE cells *in situ.* The cells also had well-defined tight junctional complexes. Foot processes on the basal side and numerous microvilli on the apical surface were also seen in the RPE cells on day 14 (Fig. 1C). RPE65, a marker for polarized RPE cells, was also detected by Western blot analysis on day 14 (Fig. 1I). Thus, our morphological and Western blot analyses showed that RPE cells grown on Transwell filters were polarized on day 14.

On the other hand, non-polarized RPE cells appeared spindle- to cobblestone- shaped (Fig. 1D), and the fine strands at the cell borders were weakly stained for ZO-1 (Fig. 1E). The expression of RPE65 was weak, and α-smooth muscle actin was expressed in these cells. These findings indicate that the RPE cells were not polarized (Fig. 1I). In addition, MCT1 was localized at the apical membrane, and laminin was located on the basolateral side of polarized RPE cells, but the distribution of these two proteins was not polarized in non-polarized RPE cells (Fig. 1J).

The TER of the RPE cells on Transwell filters was measured on day 3 and weekly for 8 weeks thereafter. The TER gradual increase from 176.7±4.5 Ω·cm^2^ on day 3 to 353.2±9.7 Ωcm^2^ on day 14, and reached a plateau at around 321.5±7.7 Ω·cm^2^ at one month (Fig. 1H).

### VEGF Secretion and TUNEL Assay

The incubation media from the upper and lower chambers of 3 experiments were used to measure the amount of VEGF-A secreted into the upper and lower chambers on days 3 and 14 after the seeding (Fig. 1G). On day 3, the amount of VEGF-A secreted from the apical surface was 110.8±8.5 pg/well, and it was 132.0±6.2 pg/well from the basal surface (*P* = 0.11, paired *t* test). On day 14, the amount of VEGF-A secreted from the apical surfaces was 265.0±7.82 pg/well, and it was 1385.9±111.5 pg/well from the basal surface (*P*<0.01, paired *t* test).

**Figure 2:**
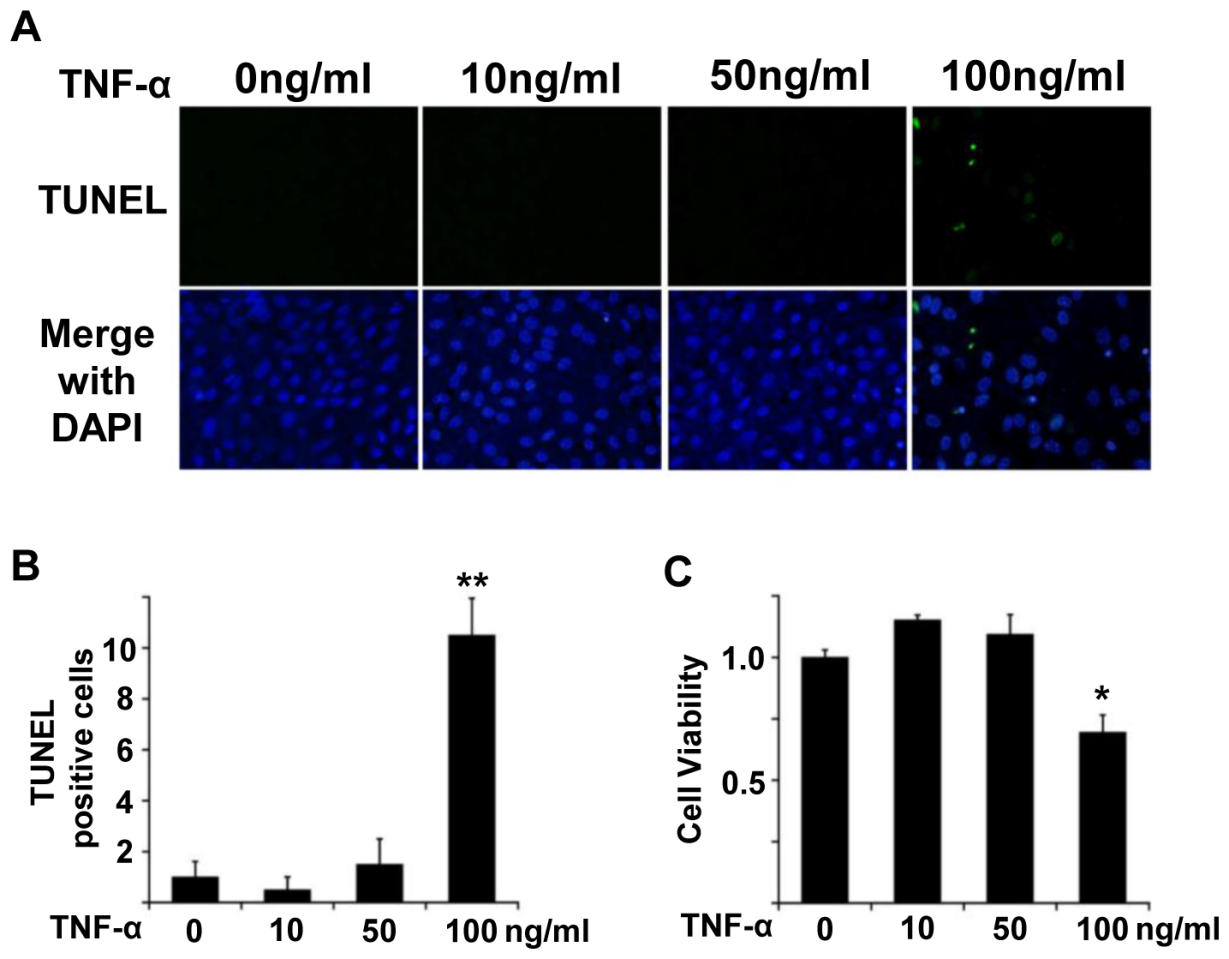
TUNEL and cytotoxic assays. A: B: The number of TUNEL positive cells/field after exposure to 50 ng/ml of TNF-α is not significantly different from that in controls, but the number was significantly higher in cells treated with 100 ng/ml TNF-α (*P*<0.01). Original magnifications, x40. C: MTT colorimetric assay did not show any significant cytotoxicity of TNF-α on RPE cells than that of controls at concentrations less than 50 ng/ml. The ratio of viable cells to control is plotted. *; *P*<0.05, **; *P*<0.01, Student’s *t* tests.

Next, we determined the cytotoxic effect of TNF-α on polarized-RPE cells by TUNEL staining. The number of TUNEL-positive polarized RPE cells/field did not differ significantly from that of controls after exposure to 50 ng/ml of TNF-α, but it was significantly higher in cells treated with 100 ng/ml TNF-α (*P*<0.01; Fig. 2A and 2B). The MTT colorimetric assay showed that the degree of cytotoxicity of TNF-α at concentrations less than 50 ng/ml were not significant (*P*<0.05; Fig. 2C). These findings were similar to the results of non-polarized RPE cells (data not shown). Thus, 10 ng/ml of TNF-α was used in the following experiments.

### Effect of TNF-α on VEGF Secretion in Polarized and Non-polarized RPE Cells

**Figure 3:**
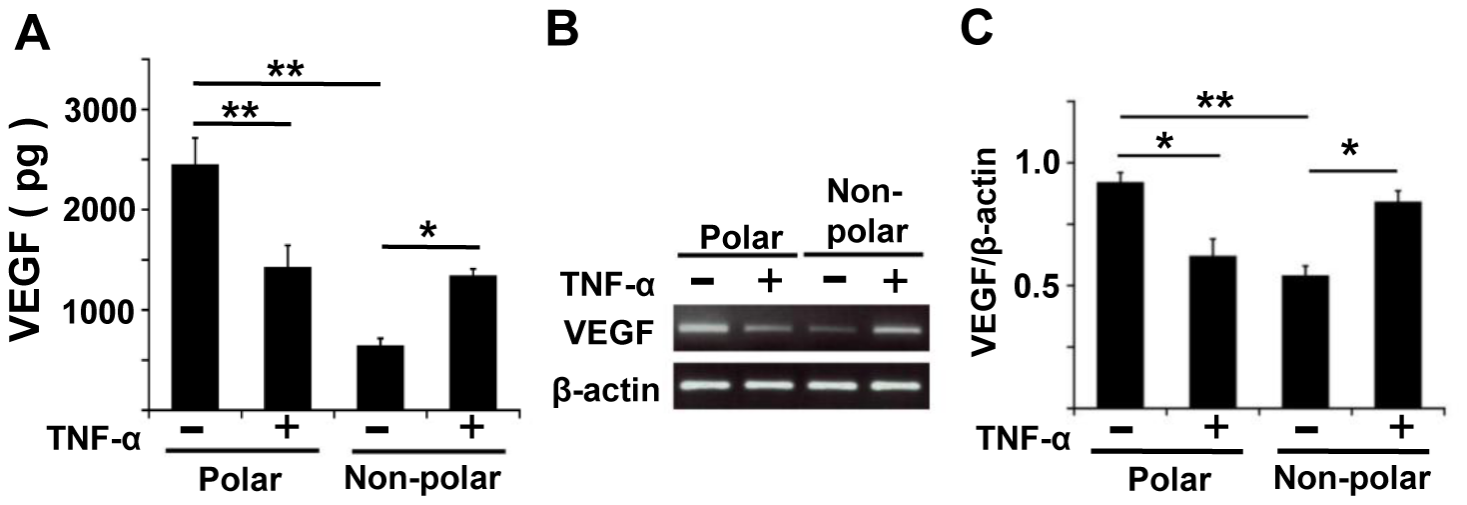
Effect of TNF-α on VEGF secretion in polarized and non-polarized RPE cells. A: In controls, VEGF was secreted at higher levels in polarized RPE than in non-polarized RPE (*P*<0.01). TNF-α significantly decreased the VEGF secretion in polarized RPE cells (*P*<0.01), but increased the VEGF secretion in non-polarized RPE cells (*P*<0.05). B: Semi-quantitative PCR shows that the expression of the mRNA of VEGF in polarized RPE cells is significantly higher than that in non-polarized RPE cells (*P*<0.01) with no TNF-α. TNF-α significantly increases the VEGF expression in non-polarized RPE cells (*P*<0.05) and decreases it in polarized RPE cells (*P*<0.05). C: Changes in the VEGF levels are expressed as ratios of the normalized levels to the level of β-actin. *; *P*<0.05, **; *P*<0.01, post hoc Tukey-Kramer test.

In controls without exposure to TNF-α, the level of VEGF was 3.8 times higher in polarized RPE cells than in non-polarized RPE cells (*P*<0.01; Fig. 3A). Although the level of VEGF was significantly lower by about 40% in polarized RPE cells (*P*<0.01), TNF-α still significantly increased the VEGF secretion in non-polarized RPE cells (*P*<0.05). These changes occurred in a dose dependent manner between 0.1 to 10 ng/ml of TNF-α (data not shown).

To determine whether the stimulation of VEGF secretion by TNF-α occurred at the transcriptional and/or translational level, the amount of the mRNA of VEGF was quantified by semi-quantitative RT-PCR. Consistent with the results of ELISA, the expression of the mRNA of VEGF in polarized RPE cells not exposed to TNF- α was significantly higher than that in non-polarized RPE cells (*P*<0.01; Fig. 3B and 3C). Exposure of the RPE cells to TNF-α significantly increased the expression of the mRNA of VEGF in non-polarized RPE cells (*P*<0.05), but decreased it significantly in polarized RPE cells (*P*<0.05; Fig. 3B and 3C).

### Subretinal TNF-α Reduces Choroidal Thickness and VEGF Expression in Rats

**Figure 4:**
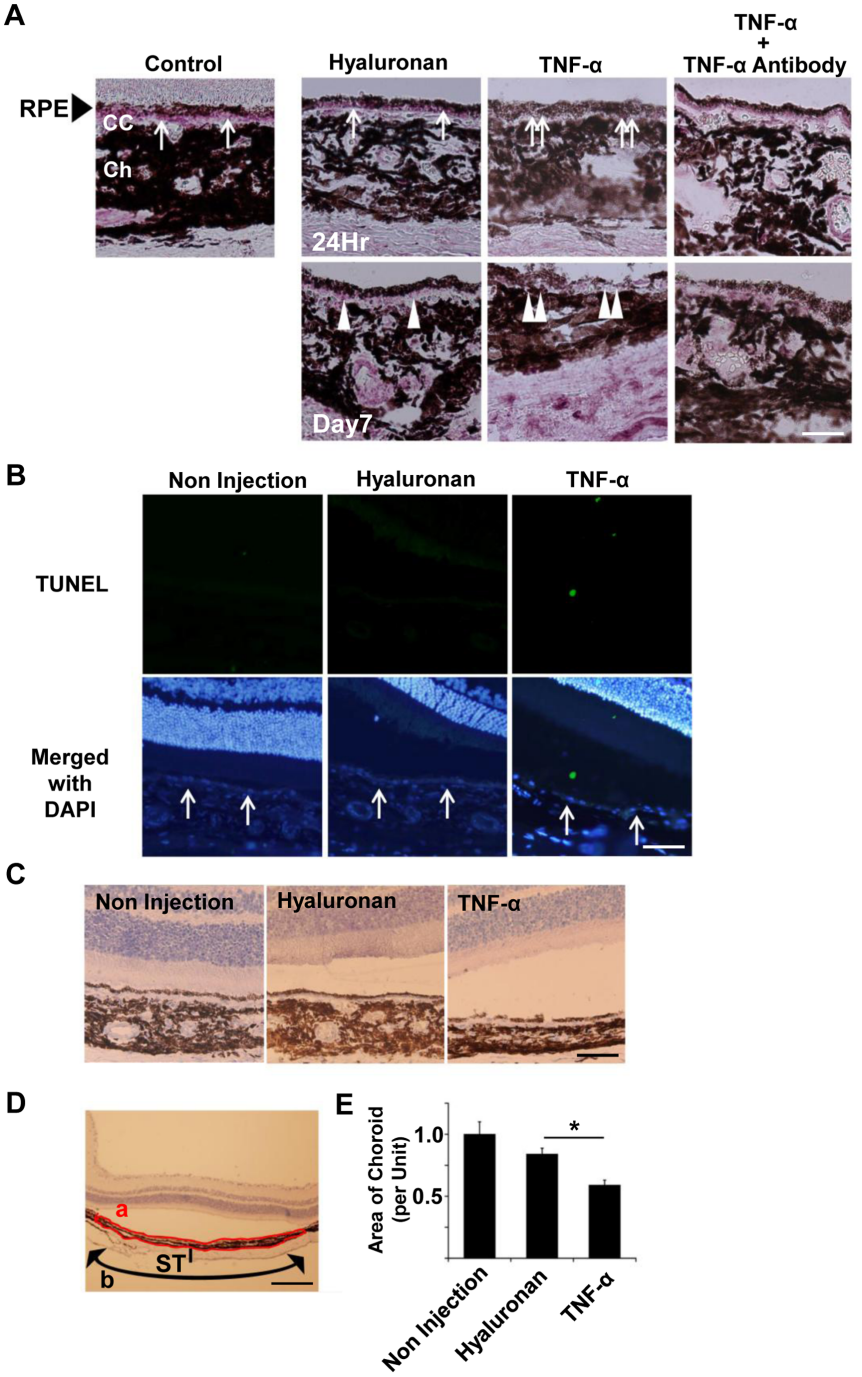
Choroidal thickness and VEGF expression in rats after subretinal injection of TNF-α. A: In controls, RPE cells are VEGF-positive especially on the basal side (arrow). After hyaloronan injection, VEGF was also found in the RPE layer (arrow). In TNF-α-injected eyes, VEGF was faint or negative in RPE cells (double arrows). On day 7, VEGF was observed in RPE in hyaloronan injected eyes (arrowhead). The RPE layer and choriocapillaris appear irregular and discontinuous in the TNF-α-injected eye. The expression of VEGF beneath the RPE was indistinct (double arrowheads). This effect was cancelled by injecting an anti-TNF-α antibody together with TNF-α. cc; choriocapillaris; Ch, choroid. Scale bar; 20 μm. B: TUNEL staining showed several positively-stained cells in the detached retina, but not the RPE cells (arrows) with or without TNF-α. Scale bar; 50 μm. C: D: E: The relative area of the choroid in a TNF-α injected eye is significantly smaller than that in hyaluronan injected or non-injected eyes. (*; *P*<0.05, vs hyaluronan injection group, post hoc Tukey-Kramer test). a: measured area of the choroid, ST: scleral thickness, b: the area of retinal detachment. Scale bar C; 50 μm, D: 200 μm.

To confirm the effect of TNF-α on the RPE cells and choroid in vivo, TNF-α was injected subretinally in a rat retinal detachment model using our earlier method [31]. The expression of VEGF was determined by immunohistochemistry in retinal sections. After 24 hours, the RPE cells in controls were stained positively for VEGF especially on the basal side (Fig. 4A, arrow). In the TNF-α-injected eyes, the VEGF staining was faint or not detected in the RPE (Fig. 4A, double arrows). On day 7 after TNF-α exposure, VEGF was not observed in the RPE cells, choriocapillaris, and choroid (Fig. 4A, double arrowhead). The RPE cell layer and choriocapillaris appeared irregular and discontinuous in the TNF-α-injected eyes as reported [33]. These effects were reduced when TNF-α was injected together with an anti-TNF-α antibody.

TUNEL staining showed cells stained positively in the detached retina but not among the RPE cells before or after exposure to TNF-α (Fig. 4B). In addition, the area of choroid/unit in the TNF-α injected eyes was significantly smaller than that in hyaluronan-injected and non-injected eyes (*P*<0.05, vs hyaluronan injection group; Fig. 4C to 4E). These results suggest that TNF-α induced morphological changes of the RPE cells and choroid together with a reduction of VEGF expression.

### JNK and NF-κB in Polarized and Non-polarized RPE Cells

To examine the intracellular signaling pathways of the TNF-α-mediated VEGF secretion, we focused on two signal pathways downstream of the TNF-α receptors; the JNK pathway and the NF-κB pathway. Both pathways are well known to be key pathways for VEGF secretion in RPE cells [16], [17], [21]. We examined the roles played by these pathways on the TNF-α-mediated VEGF secretion with different inhibitors of specific pathways.

**Figure 5:**
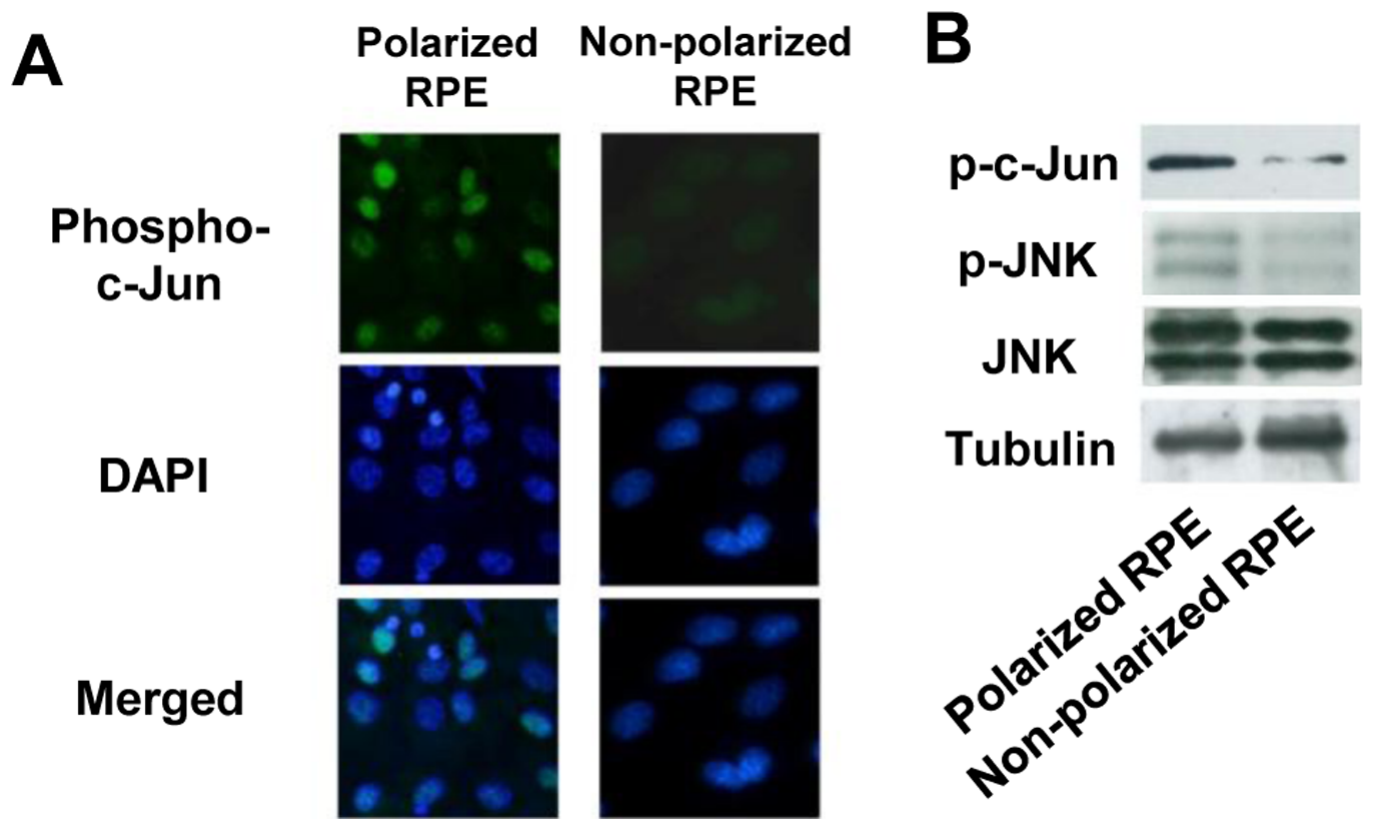
JNK expression in polarized and non-polarized RPE cells. A: Immunofluorescein staining for phospho-c-Jun. In controls, immunohistochemistry shows that phospho-c-Jun is expressed more strongly in polarized RPE cells than that in non-polarized RPE cells. B: Western blot shows that phospho-JNK and phospho-c-Jun are expressed more strongly in polarized RPE than non-polarized RPE. JNK is expressed comparably in both types of RPE cells. Original magnifications x200.

First, the expression of JNK was examined in polarized and non-polarized RPE cells by immunohistochemistry and Western blotting. In controls, immunohistochemistry showed that phospho-c-Jun, which is regulated by JNKs, was expressed at higher levels in polarized RPE cells than that in non-polarized RPE cells (Fig. 5A). Western blot also showed that phospho-JNK and phospho-c-Jun were expressed significantly more strongly in polarized RPE than in non-polarized RPE cells (Fig. 5B). On the other hand, JNK was expressed at about the same level in both types of RPE cells.

**Figure 6:**
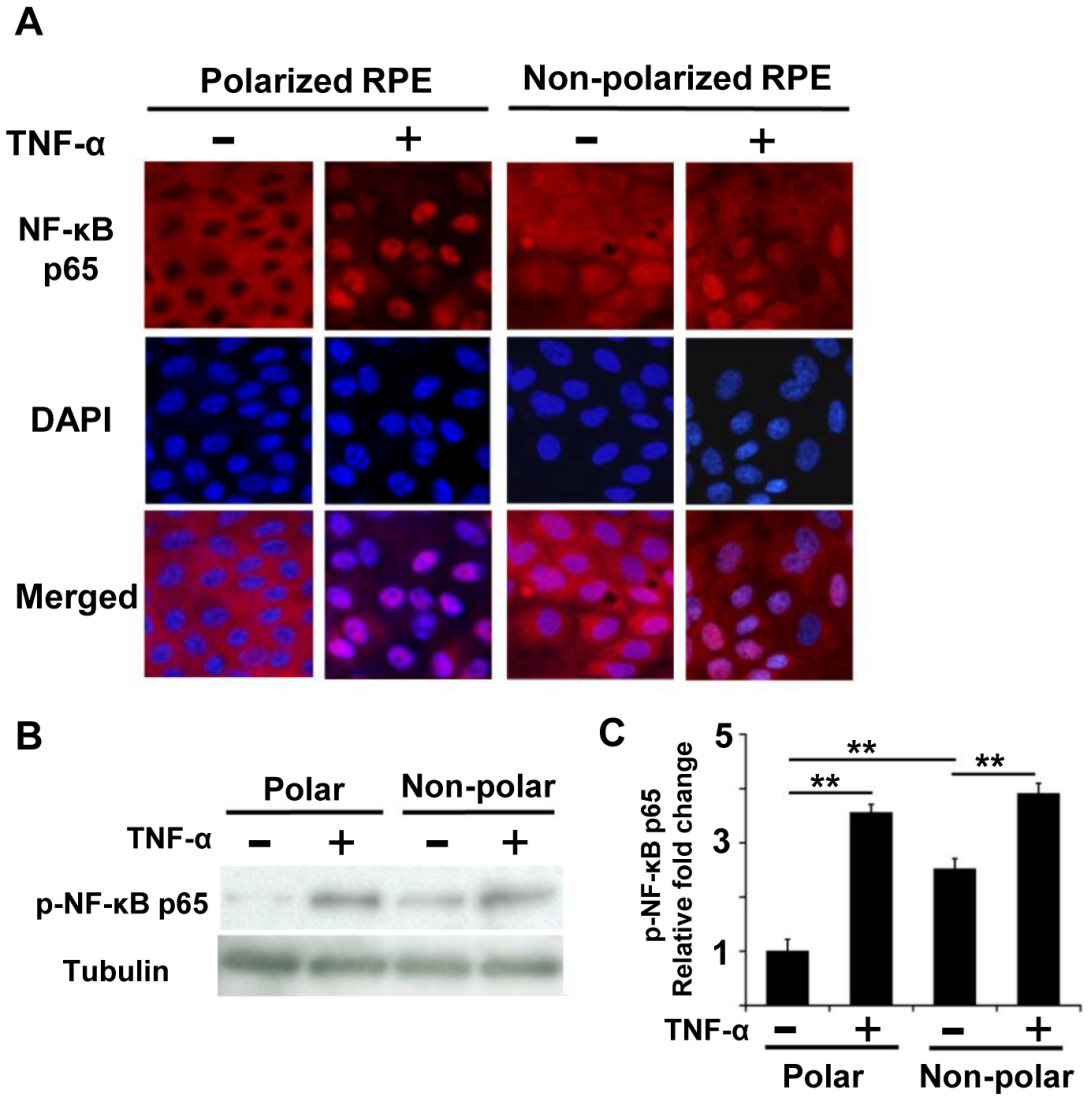
Expression of NF-κB in polarized and non-polarized RPE cells. A: In controls without TNF-α, immunohistochemistry shows that NF-κB p65 is expressed mainly in the cytosol of polarized RPE cells, while it is seen in both the cytosol and nucleus in non-polarized RPE cells. After TNF-α exposure, NF-κB p65 is expressed almost exclusively in the nucleus of polarized RPE cells, but NF-κB p65 is expressed mainly in the nucleus of non-polarized RPE cells. B: In Western blot analysis, a faint phospho-NF-κB p65 band can be seen in the polarized RPE cells without exposure of TNF-α, but it increases significantly after exposure to TNF-α. In contrast, phospho- NF-κB p65 is detected in non-polarized RPE cells without TNF-α, and it is increased after exposure to TNF-α. C: Semi-quantification of these band adjusted by control band of tubulin. The expression of phospho-NF-κB p65 in non-polarized RPE is greater than that in polarized RPE (*P*<0.01). Although TNF-α increased the phospho-NF-κB p65 by 1.5 times in non-polarized RPE cells (*P*<0.01), it was increased by more than 3 times in polarized RPE cells (*P*<0.01). *; *P*<0.05, **; *P*<0.01, post hoc Tukey-Kramer test. Original magnifications, x200.

Next, we examined the level of NF-κB expression. In controls without TNF-α, immunohistochemistry showed that NF-κB p65 was mainly expressed in the cytosol of polarized RPE cells, but it was seen in both the cytosol and nucleus in non-polarized RPE cells (Fig. 6A). After TNF-α exposure, NF-κB p65 was detected mainly in the nucleus in polarized RPE cell (Fig. 6A), and weakly in the nucleus in non-polarized RPE cells. Interestingly, some NF-κB p65 was still observed in the cytosol even after exposure to TNF-α in non-polarized RPE cells (Fig. 6A).

In Western blot analyses, a weak phospho- NF-κB p65 band was detected in polarized RPE cells without TNF-α, but the signal was significantly increased after exposure to TNF-α. In contrast, phospho- NF-κB p65 was detected in non-polarized RPE cells without TNF-α, and it was also increased after TNF-α (Fig. 6B). A semi-quantification of these band corrected by the intensity of the control band of tubulin showed that the expression of phospho-NF-κB p65 in non-polarized RPE was significantly stronger than that in polarized RPE (*P*<0.01; Fig. 6C). Although TNF-α increased the expression of phospho-NF-κB p65 by 1.5 times in non-polarized RPE cells (*P*<0.01; Fig. 6C), it was increased by more than 3 times in polarized RPE cells (*P*<0.01; Fig. 6C).

### Differences of VEGF Secretion by Cell Polarity associated with JNK and NF-kB Pathways

**Figure 7:**
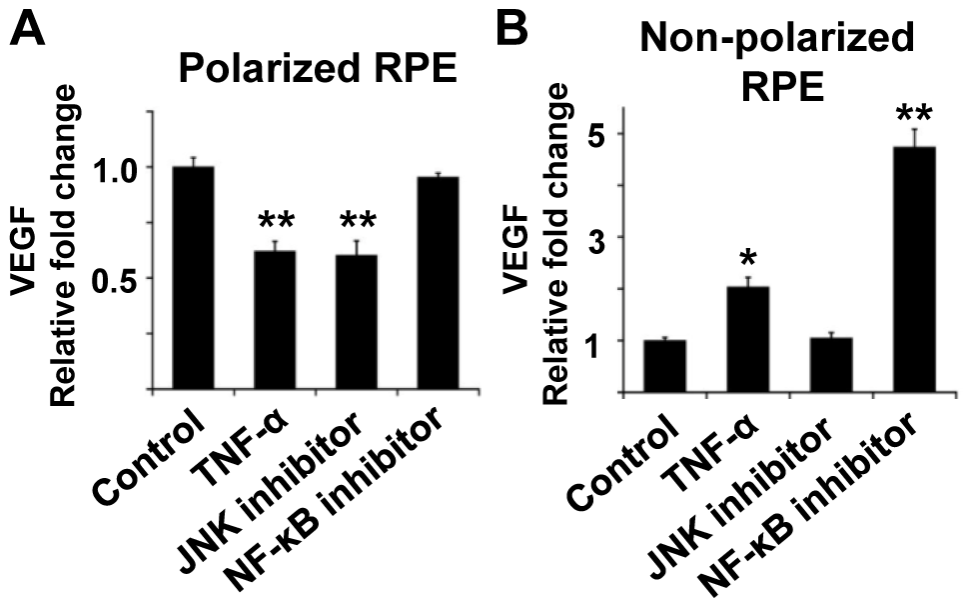
Differences in VEGF secretion by cell polarity associated with JNK and NF-kB pathways. A: VEGF secretion in polarized RPE cells. TNF-α decreases the secretion of VEGF by about 40% compared to that of controls (*P*<0.01). JNK inhibitor, SP600125, significantly decreases the VEGF secretion by 40% (*P*<0.01). CAPE, a NF-κB inhibitor, does not have any significant effect on the degree of VEGF secretion. B: VEGF secretion in non-polarized RPE cells. TNF-α increases the secretion of VEGF more than that in the controls (*P*<0.05). SP600125 alone does not have any significant effect, but CAPE alone significantly increases the VEGF secretion by almost five times (*P*<0.01). *; *P*<0.05, **; *P*<0.01, student’s *t* tests.

In polarized RPE cells, TNF-α decreased the secretion of VEGF by about 40% compared to that of controls (*P*<0.01; Fig. 7A). The JNK inhibitor, SP600125, significantly decreased the VEGF secretion by 40% (*P*<0.01; Fig. 7A). CAPE, a NF-kB inhibitor, did not have any significant effect on the degree of VEGF secretion.

In non-polarized RPE cells, TNF- α increased the secretion of VEGF greater than that in the controls (*P*<0.05; Fig. 7B). SP600125 alone did not have any significant effect, but CAPE alone significantly increased VEGF secretion by almost five times (*P*<0.01; Fig. 7B).

The involvement of the p38 MAPK, Erk, and AP-1 in the VEGF secretion pathways was evaluated because they are known to be important pathways in RPE cells [21], [34]. Inhibition of the p38 MAPK, Erk, or AP-1 pathways significantly decreased the secretion of VEGF in both non-polarized and polarized RPE cells *(P*<0.01 both; Figure S1). In polarized RPE cells, inhibitors of the p38 MAPK, Erk, and AP-1 pathways had no additional effect on the reducing the effect of TNF-α. In non-polarized RPE cells, the inhibitors of the p38 MAPK, Erk, and AP-1 pathways reduced the increasing effect of TNF-α. Therefore, inhibition of p38 MAPK, Erk, and AP-1 reduced the VEGF secretion but its pattern was not changed by the polarization of the cells.

### Sustained JNK Pathway Phosphorylation by TNF-α in NF-kB Inhibited RPE Cells

The results of recent studies showed that the JNK and NF-κB pathways play important roles in the secretion of VEGF, and that a crosstalk between NF-κB and JNK pathways is a major determinant of the fate of cells during a TNF-α challenge [35]–[37]. This was also found in cases of cancer cells in which the NF-κB pathway inhibits the activation of the JNK pathway [38].

**Figure 8:**
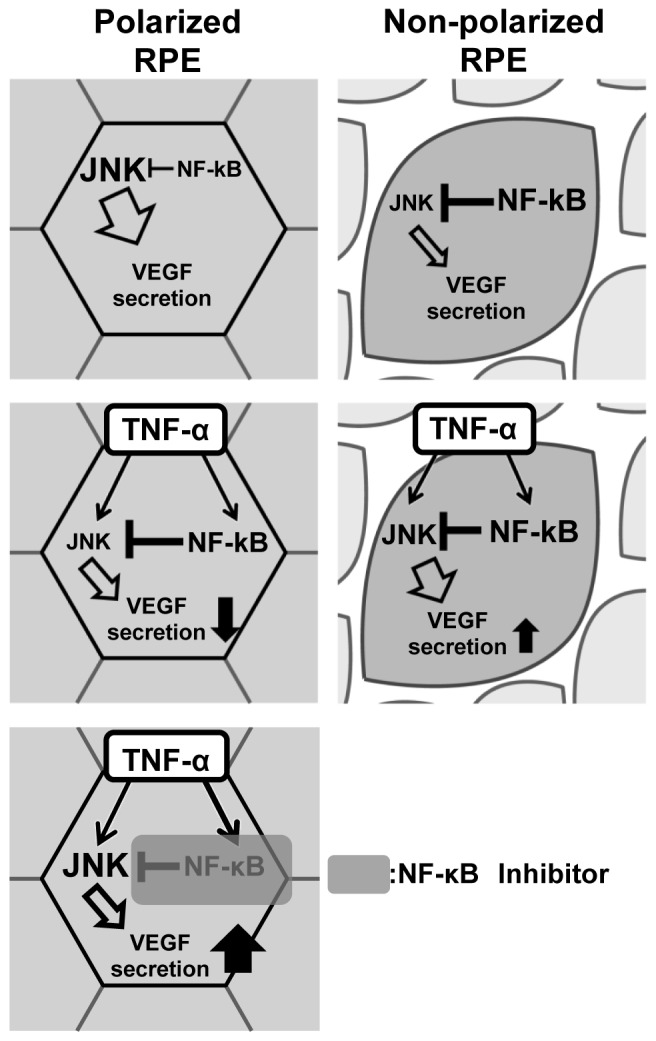
Schematic drawing illustrating the effects of TNF-α exposure on polarized and non-polarized RPE cells. In polarized RPE cells, JNK is activated, and NF-κB is inactivated which results in minimal inhibitory effect on JNK. Thus, VEGF secretion is high (top left). After TNF-α exposure, both the JNK and NF-κB pathways are activated resulting in marked increases of inhibitory effect of NF-κB on JNK. Thus, VEGF secretion is comparatively inhibited (middle left). In NF-κB being inhibited, its inhibitory effect on JNK by TNF-α could not work, either. Therefore, VEGF secretion is reversely increased (bottom left). In non-polarized RPE cells, NF-κB is strongly activated which results in a strong inhibitory effect on JNK. Thus, JNK is inactivated and VEGF secretion is low (top right). After TNF-α exposure, both JNK and NF-κB are activated but the additional activation of NF-κB by TNF-α is limited. Thus, JNK is comparatively increased with increased VEGF secretion (bottom right).

**Figure 9:**
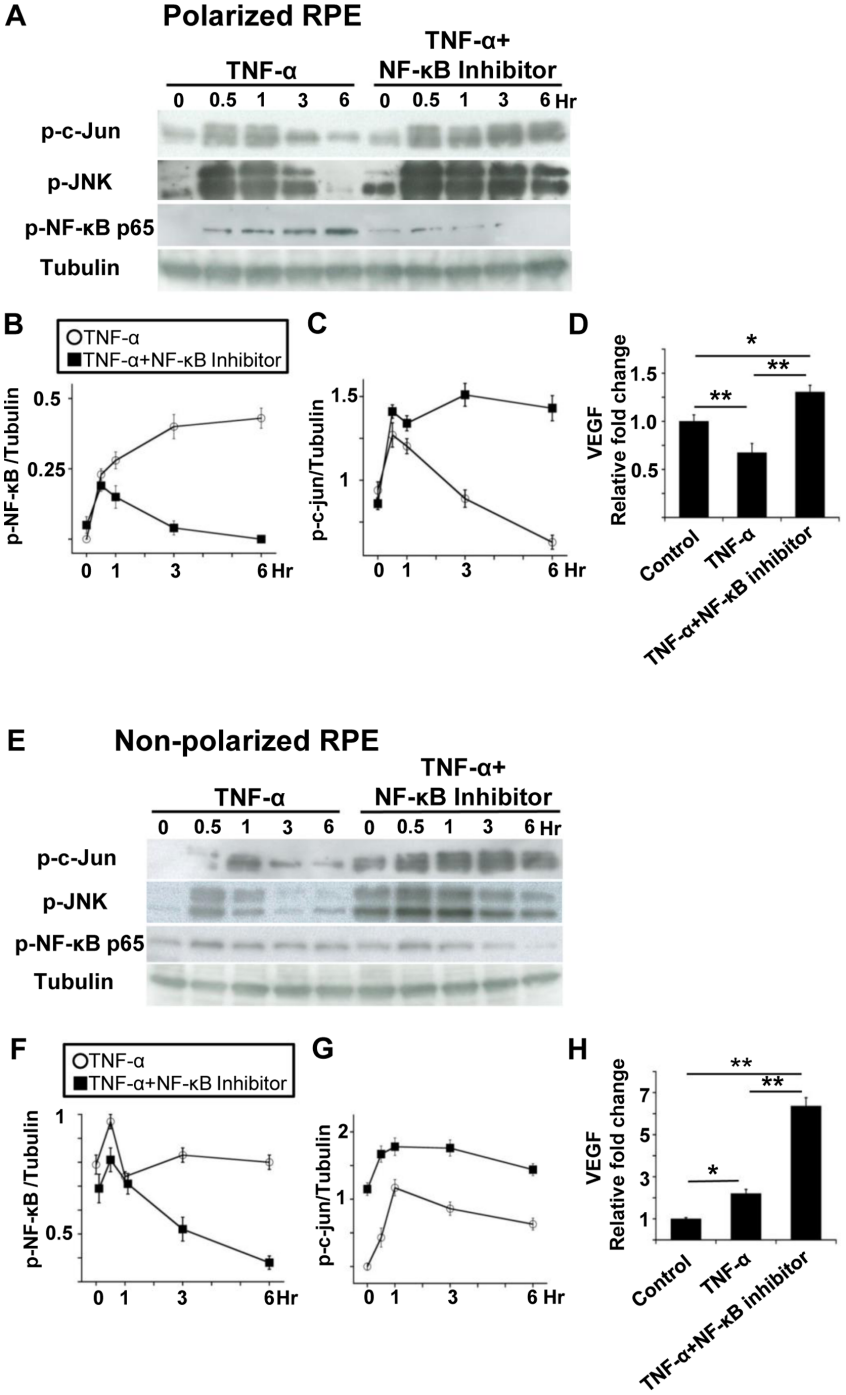
Sustained JNK pathway phosphorylation by TNF-α in NF-κB-inhibited RPE cells. A: Western blot analysis of polarized RPE cells for phospho-c-Jun, phospho-JNK, and phospho NF-κB p65. B: Chronological change of p-NF-κB p65. Exposure to TNF-α leads to an activation NF-κB p65 at 30 minutes, and the increase is sustained for 6 hours. C: Chronological change of p-c-Jun. Although phospho-c-Jun is decreased at 3 hours after TNF-α exposure, it is still high after 6 hours with CAPE. The phospho-c-Jun level was 40% lower at 6 hours than time 0 after TNF-α exposure. D: Concentration of VEGF. The level of VEGF lower than that of the controls after TNF-α exposure (*P*<0.01) and higher than that of controls after TNF-α with CAPE (*P*<0.05). E: Western blot of non-polarized RPE cells for phospho-c-Jun, phospho-JNK, and phospho NF-κB p65. F: Chronological change of p-NF-κB p65. Although NF-κB p65 is already activated before TNF-α exposure, it was slightly activated at 30 minutes and sustained for 6 hours. G: Chronological change of phospho-c-Jun. Although phospho-c-Jun is decreased at 3 hours with TNF-α alone, it remains high even at 6 hours with CAPE. Comparing the phospho-c-Jun level, it was 3 times higher at 6 hours than at time 0 after TNF-α exposure. Pretreatment with CAPE increases the phospho-c-Jun level 5 times higher than that of controls, and it is further increased by 40% after 6 hours. H: Concentration of VEGF. VEGF concentration increases 2 times higher than that of controls after TNF- α exposure and 6 times higher than that of controls after TNF- α and CAPE (*P*<0.05 and *P*<0.01). This changing is similar to that of phospho-c-June at 6 hour (E). *; *P*<0.05, **; *P*<0.01, post hoc Tukey-Kramer test.

Therefore, we hypothesized that this crosstalk would lead to opposite responses of VEGF secretion between polarized and non-polarized RPE cells (Fig. 8). We then examined effect of TNF-α on JNK phosphorylation with and without CAPE, a NF-κB inhibitor, in polarized and non-polarized RPE cells. In polarized RPE cells, exposure to TNF-α led to an activation NF-κB p65 at 30 minutes which was sustained for 6 hours (Fig. 9A and 9B). It also activated c-Jun which peaked at 30 minutes and it gradually decreased for 6 hours (Fig. 9A and 9C). On the other hand, TNF-α with CAPE activated c-Jun, and the activation was sustained even after 6 hours (Fig. 9A and 9C). Although the level of phospho-c-Jun decreased from 3 hours after the TNF-α exposure, it was still high after 6 hours with CAPE (Fig. 9C). The phospho-c-Jun level was 40% lower at 6 hours than at time 0 after the TNF-α exposure. The concentration of VEGF was lower than that of the controls after TNF-α exposure (*P*<0.01; Fig. 9D) and higher than the controls after TNF-α with CAPE (*P*<0.05; Fig. 9D). The level then corresponded to the phospho-c-Jun value.

Exposure of non-polarized RPE cells to TNF-α led to the activation of c-Jun which peaked at 1 hour and then gradually decreased for 6 hours. TNF-α with CAPE, on the other hand, also activated the kinase and sustained the activation for 6 hours (Fig. 9E). Although NF-κB p65 was already activated before the TNF-α exposure, it was slightly activated at 30 minutes and sustained for 6 hours (Fig. 9F). The level of phospho-c-Jun was decreased at 3 hours with TNF-α alone, but it remained high even at 6 hours with CAPE (Fig. 9G). Compared to the phospho-c-Jun level, it was 3 times higher at 6 hours than at time 0 after TNF-α exposure. Pretreatment with CAPE increased the phospho-c-Jun level 5 times higher than that in the controls, and it was further increased by 40% after 6 hours (Fig. 9G). The increase in the VEGF concentration was 2 times higher than that of the control after the TNF-α exposure and 6 times higher than that of the control with TNF-α and CAPE (*P*<0.05 and *P*<0.01, respectively; Fig. 9H).

This changing pattern was similar to that of phospho-c-Jun (Fig. 9E). The same pattern was observed after phospho-JNK antibody both in polarized and non-polarized RPE cells.

## Discussion

Our immunohistochemical *in situ* studies showed that VEGF was expressed predominantly on the basal side of the RPE cells in normal rats. However, this expression became weak 24 hours after a subretinal injection of TNF-α. After 7 days, the choroidal thickness was reduced significantly compared to that of the control, and the expression of VEGF was absent in the RPE cells. Because TNF-α is a strong inducer of apoptosis, these changes might be primarily caused by a direct apoptosis-inducing effect on the RPE cells. However, apoptotic RPE cells were not observed by the TUNEL method. The findings that subretinal TNF-α induced atrophy of the RPE and choroid are consistent with an earlier report [33]. More importantly, these changes were associated with a reduction of VEGF expression by RPE cells. Although the reduction of VEGF might not necessarily be the most critical factor causing the choroidal atrophy, it is highly likely that the reduction of VEGF plays some role in the atrophy of the choriocapillaris.

It was reported that TNF-α or other inflammatory cytokines augmented the secretion of VEGF from RPE cells in vitro [16], [17], [21]. Histological studies of surgically resected CNV membrane showed that RPE cells in the membranes expressed VEGF, which suggest that the RPE cells play a role in the formation of CNV by secreting VEGF [13]–[15]. We found that TNF-α decreased the secretion of VEGF in polarized RPE cells but increased it in non-polarized RPE cells. This opposing action of TNF-α on VEGF secretion by RPE cells has not been reported before. We suggest that a crosstalk of the signal transduction system was a key factor for these observations.

The JNK and NF-κB pathways play important roles in the secretion of VEGF [16], [17], [21], [34], [39], [40], [41], and a crosstalk between the JNK and NF-κB pathways is a major determinant of the fate of cells where NF-κB inhibits the activation of JNK [35], [37], [38]. Therefore, we hypothesized that this crosstalk is responsible for the opposite response patterns of VEGF secretion between polarized and non-polarized RPE cells (see Fig. 8).

Without TNF-α exposure, NF-κB was not activated but JNK was activated in polarized RPE cells. In contrast, NF-κB was highly activated but JNK was inactivated in non-polarized RPE cells (Table 1). Inhibition of NF-κB resulted in JNK activation in non-polarized RPE cells. The increases in the JNK activity was higher in non-polarized RPE cells than in polarized RPE cells probably because in polarized RPE, the baseline NF-κB activity is already low and the inhibition of NF-κB by CAPE does not result in any major effect (Table 1). Taken together, these results indicate that there is a JNK pathway in RPE cells which can be inhibited by NF-κB.

**Table:** Table 1. Effect of VEGF secretion on JNK and NF-κB.

	Polarized RPE			Non-polarized RPE		
	Control	TNF-α	TNF-α with CAPE	Control	TNF-α	TNF-α with CAPE
**NF-κB activity**	**–**	**+**	**–**	**++**	**++**	**+**
**JNK activity**	**++**	**+**	**+++**	**–**	**+**	**++**
**VEGF secretion**	**++**	**+**	**+++**	**+**	**++**	**+++**

CAPE: Caffeic Acid Phenethyl Ester, NF-κB Inhibitor.

The findings after TNF-α exposure also support the hypothesis of crosstalk between JNK, NF-κB, and VEGF. TNF-α activated both the JNK and NF-κB pathways. In polarized RPE cells, JNK was already activated and NF-κB was inactivated, thus activation of NF-κB became significant after TNF-α exposure compared to control leading to inhibition of JNK and down-regulation of the VEGF expression (Fig. 8). In non-polarized RPE cells, the JNK activity is low and NF-κB is already highly activated, thus the additional activation of NF-κB by TNF-α was limited and the stimulatory effect of TNF-α on JNK predominates leading to an increase in the secretion of VEGF compared to controls. This crosstalk system can explain the opposite VEGF secretion responses to TNF-α between polarized and non-polarized RPE cells (Fig. 8).

Similar observations that TNF-α up-regulates VEGF in dedifferentiated/pathologic chondrocytes but TNF-α down-regulates VEGF in differentiated/physiological chondrocytes have been reported by Honorati et al [42]. Thus, these findings may not be limited to RPE cells and could be applicable to other systems where VEGF is critical in physiological and pathophysiological processes.

At present, it has been assumed that inflammatory cytokines up-regulate VEGF in the RPE cells from the results of in vitro studies. However, most of these studies used non-polarized RPE cells [16], [17], [21], which may account for the role of RPE in the wet type AMD. Our results showed that the molecular mechanism for the down-regulation of VEGF in RPE cells by inflammation could lead to the dry type of AMD with choroidal atrophy. Considering the similarity of polarized RPE cells to the *in vivo* condition, this may better reflect the actual pathophysiology of RPE cells. In addition, our results indicate that NF-κB is tonically activated in cultured cells. Because growth on a plastic dish is not very physiological it was not unexpected. The results from non-polarized RPE cells may not necessarily reflect the actual *in vivo* condition, and thus these data should be interpreted carefully when trying to transfer them to the clinical conditions.

There are some limitations in this study. A gene silencing method, such as siRNA, might be a better method to determine the role of a specific molecule. However, our preliminary studies showed that the highly polarized RPE cells did not uptake sufficient amounts of siRNA by lipofection. Thus, pharmacological inhibitors were used, but it should be remembered that the pharmacological method is not always specific to a single molecule.

In addition, to conclude that the pathways we studied were the only pathways, it is necessary to determine the gene expression profile. However, this was not done. Thus, it is possible to say that the pathways studied were important, but not necessarily the most important pathways. This should be remembered when interpreting our results.

In conclusion, we found that TNF-α down-regulates VEGF secretion in polarized RPE cells but up-regulates it in non-polarized RPE cells. These results are due to the opposing activity levels of the JNK and NF-κB pathways. In certain clinical conditions, such as AMD, the RPE cell polarity changes at different stages of the disease with the RPE cells being polarized early on, and some RPE cells losing their cellular polarity at the late stages. Understanding the initial phase of a disease process is crucial for understanding the development of the disease processes. Thus, our studies using polarized RPE cells and VEGF secretion may help us understand the process of the evolution of AMD.

## Supporting Information

Figure S1 Decreased VEGF secretion by inhibiting p38 MAPK, Erk, or AP-1 pathways. Inhibition of p38 MAPK (A and B), Erk (C and D), or AP-1 (E and F) significantly decreases the secretion of VEGF in both non-polarized and polarized RPE cells *(P*<0.01, Student’s *t* tests). In polarized RPE cells, none of three inhibitors has any additional effect on the reducing the effect of TNF-α. In non-polarized RPE cells, p38 MAPK, Erk, or AP-1 inhibition reduced the increasing effect of TNF-α. Inhibition of p38 MAPK, Erk or AP-1 reduced VEGF secretion but its pattern was not changed by the polarization of the cells.
(TIF)
