# Supplementary figures and images for: TNF-α Decreases VEGF Secretion in Highly Polarized RPE Cells but Increases It in Non-Polarized RPE Cells Related to Crosstalk between JNK and NF-κB Pathways
Source: PLoS One. 2013 Jul 29;8(7):e69994. doi: 10.1371/journal.pone.0069994 (PMC3726732; doi:10.1371/journal.pone.0069994)

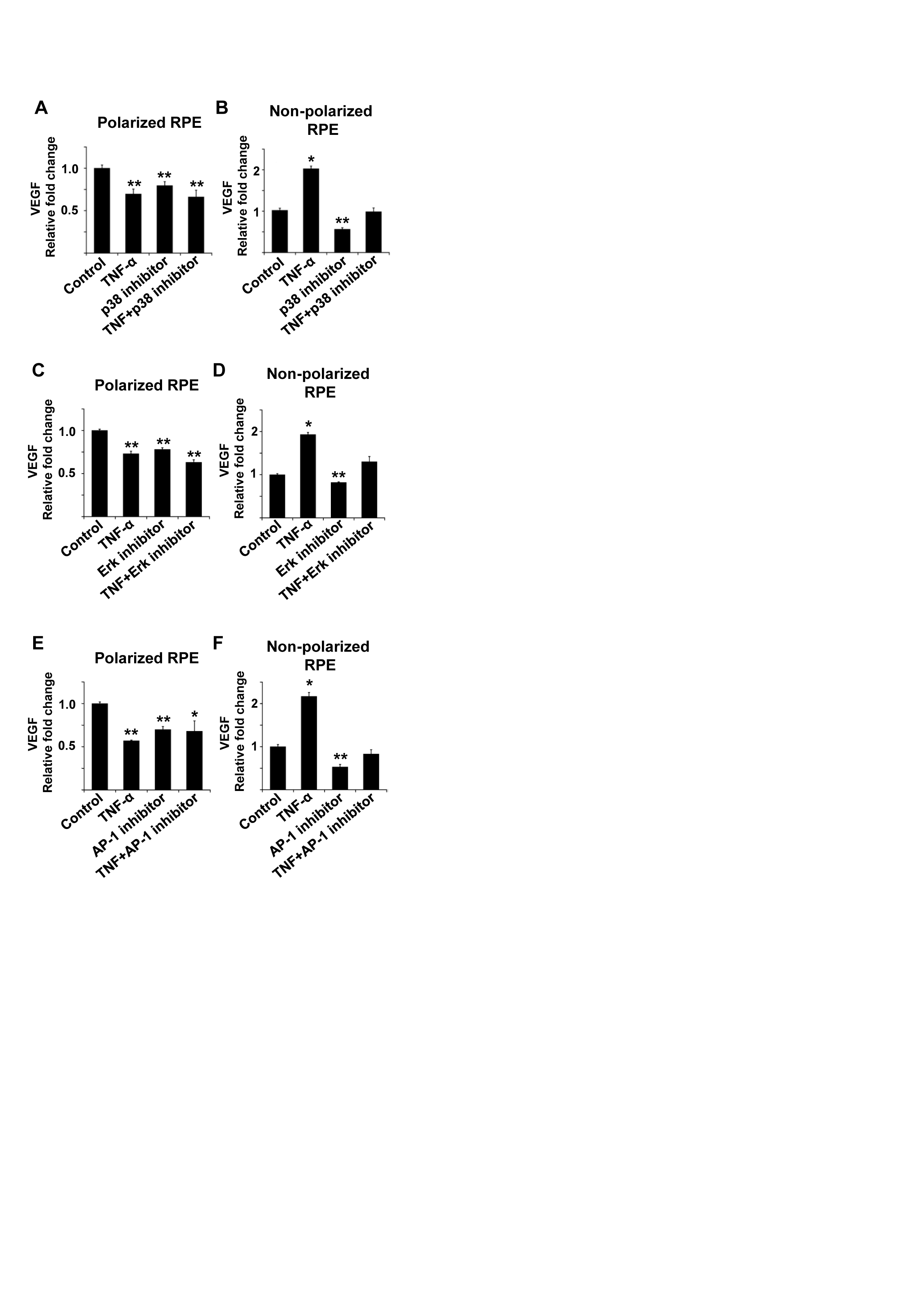

Supplement: Figure S1 — Inhibition of p38 MAPK (A and B), Erk (C and D), or AP-1 (E and F) significantly decreases the secretion of VEGF in both non-polarized and polarized RPE cells (P<0.01, Student’s t tests). In polarized RPE cells, none of three inhibitors has any additional effect on the reducing the effect of TNF-α. In non-polarized RPE cells, p38 MAPK, Erk, or AP-1 inhibition reduced the increasing effect of TNF-α. Inhibition of p38 MAPK, Erk or AP-1 reduced VEGF secretion but its pattern was not changed by the polarization of the cells. (TIF) [file pone.0069994.s001.tif]
